# Effects of emotion words activation and satiation on facial expression perception: evidence from behavioral and ERP investigations

**DOI:** 10.3389/fpsyt.2023.1192450

**Published:** 2023-07-31

**Authors:** Qiang Xu, Weihan Wang, Yaping Yang, Wanyue Li

**Affiliations:** ^1^Department of Psychology, Ningbo University, Ningbo, China; ^2^School of Psychology, South China Normal University, Guangzhou, China

**Keywords:** facial expressions, emotion concept, semantic satiation, emotion-label words, emotion-laden words

## Abstract

**Objective:**

The present study investigated the impact of emotion concepts obtained from external environmental experiences on the perception of facial expressions by manipulating the activation and satiation of emotion words, which was based on the argument between basic emotion theory and constructed emotion theory.

**Methods:**

Experiment 1 explored the effects of emotion activation on happy, disgusted, emotion-label words and emotion-laden words in a facial expression judgment task through behavioral experimentation. Experiment 2 explored the effect of semantic satiation on emotion-label words and emotion-laden words using the event-related potential technique.

**Results:**

Experiment 1 found that facial expression perception was influenced by both types of emotion words and showed a significant emotional consistency effect. Experiment 2 found that N170 exhibited a more negative amplitude in the consistent condition compared to the inconsistent condition in the right hemisphere. More importantly, in the later stage of facial expression processing, emotion-label words and emotion-laden words both obstructed the perception of disgusted facial expressions and elicited more negative N400 amplitude in the emotion consistency condition, showing a reversed N400 effect.

**Conclusion:**

These results suggested that emotion concepts in the form of language influenced the perception of facial expressions, but there were differences between happy and disgusted faces. Disgusted faces were more dependent on emotion concept information and showed different performances in semantic activation and satiation conditions.

## Introduction

How do we read the emotions of others when we see their facial muscle movements? According to Ekman's basic emotion theory, the automatic recognition of the six basic emotions—happiness, sadness, anger, fear, disgust, and surprise—involves a specific neural activity that is scarcely modified by other environmental information (1, 2). More recent studies of emotions suggest that individuals' perceptions of emotions probably develop through experience and are not entirely innate (3–5). The theory of constructed emotion proposed by Barrett holds that emotion perception relies on the construction of emotion concepts (6) and that the development of emotion concepts is linked to the individual's acquisition of emotional language. Barrett assumed that there is an internal model based on past experiences and expressed in terms of concepts (6). Concepts are the aggregation of specific stimuli that are used to predict the stimuli that will occur in the sensory environment and to initiate the most appropriate behavior. Specifically for emotion perception, the internal model shows that in the process of creating emotion concepts, individuals categorize numerous emotional stimuli from learning and experience and store them in different emotional categories, which are externalized in the form of emotion words expressed in language, such as “happy” and “fearful”. The theory of constructed emotion considers language as an important internal context that can promote, obstruct, or even shape the perception of facial expressions (7–9). Based on the debate between constructed emotion theory and basic emotion theory, exploring the role of emotion words derived from learning and experience in the perception of facial expressions is important for us to understand the process of emotion perception and the top-down role of emotion concepts expressed in language in the perception of facial expressions.

The relationship between the developing vocabulary and facial emotion perception was reviewed by Widen and Russell (4). She suggested that at an early age, children relied on a valence-based positive–negative emotional dichotomy and that it took several years of cultural and language learning before they could categorize facial expressions, especially some complex, mixed expressions, into discrete emotional categories (10). Nook et al. found that increasing verbal knowledge mediated the development of emotion representation (11). These results indicated that the acquisition of emotion vocabulary knowledge facilitated the development of multidimensional emotion perception in adulthood, beyond the dichotomous valence scaling of childhood to the consideration of other qualities. Recently, studies on the language context effect also found that individuals' emotion perception was influenced by the top-down processing of semantic information and exhibited an emotional consistency effect. When words or sentences with positive or negative emotional meanings were presented with faces as background information, emotionally consistent language context promoted the processing of facial expressions and showed shorter reaction time (RT) and higher accuracy (ACC), while emotionally inconsistent language context hindered facial expression processing (12–14).

Researchers found that the facial-sensitive N170 component located in the temporo-occipital region showed a larger amplitude when language contexts were inconsistent with facial expressions. These suggested that the early stage of face processing was influenced by language context and showed sensitivity to emotional consistency (13, 15, 16). In the later stage of face processing, the N400 component, which is associated with cognitive conflict, had also drawn the attention of researchers. N400 is mainly located in the centroparietal regions and occurs around 400 ms after the stimulus onset. Early researchers believed that N400 is mainly sensitive to semantic inconsistency. For example, a sentence's final word would elicit a more negative N400 amplitude when it did not correspond to the rest of the sentence's meaning (17). Because inconsistent scene pictures from the visual channel and inconsistencies in face and identity information could also evoke a more negative N400 component (18, 19), researchers have found that the N400 may be an index for the processing of meaningful stimuli as well as information integration (20). Compared with emotional consistency, the sequential presentation of stimuli with conflicting emotional meanings elicited more negative N400 amplitudes, regardless of whether the target stimuli were faces (13, 16) or words (21).

The existing studies showed that linguistic information played an important role in facial expression processing and affected neurocognitive activity. However, most previous studies had focused on the state of emotional priming induced by emotional semantic activation, while there was still less research on whether and how emotion words obstructed facial expression processing when there was semantic satiation of emotion words, making it difficult to verify the existence of a direct link between emotional semantics of words and facial expression perception.

Individuals who look at or repeatedly read words for extended periods of time may become unable to effectively identify the meaning of words because their ability to extract the meaning of words is temporarily reduced. In the 1960s, researchers detected this phenomenon and named it semantic satiation (22). In an event-related potential (ERP) study that explored semantic satiation, Kounios et al. examined the stages of semantic satiation using the N400 component as an index (23). Priming words were presented in the visual and auditory channels, respectively, and the participants were required to make semantic judgments about the target words and compare the N400 wave amplitude differences between the relevant and irrelevant word conditions. Semantic satiation occurred after 30 repetitions of the priming words in both visual and auditory channels, resulting in a reduction of the N400 difference wave between the relevant and irrelevant conditions. This study suggested that semantic satiation affected the processing of semantic information and that semantic satiation could occur in different sensory channels. Ströberg et al. further verified that the N400 amplitude decreased under the condition of semantic satiation resulting from 30 times the priming word presentation (24). Moreover, by varying the features of the priming words (e.g., font, letter case), they found that the change in N400 amplitude resulted from the satiation of semantic processing rather than the satiation of the structural features of the priming words. Previous studies have shown that the processing of word meaning was obstructed in the semantic satiation condition, resulting in diminished cognitive conflict in the inconsistency condition, thus showing a decreased N400 effect.

It was found that the semantic satiation of emotional words affects the perception of subsequent emotional stimuli (25, 26). Lindquist et al. explored the relationship between language and emotion perception in three experiments (25). In Experiments 1 and 2, facial expression pictures were presented after participants had repeatedly read emotion-label words 3 or 30 times; participants were then required to make emotion consistency judgments of word and face emotion. Participants showed longer RTs after semantic satiation. These experiments suggested that emotional semantic satiation obstructed individuals' judgments of face emotion categories and even caused difficulties in perceiving facial expressions. Experiment 3 explored the effect of semantic satiation of emotion-label words on the ACC of face emotion recognition. The ACC of face emotion categorization decreased in the semantic satiation condition (25). Lindquist et al.'s study found that semantic satiation of emotion-label words obstructed individuals' perception of the same type of emotional faces, suggesting that emotional language was involved in the processing of facial expressions even though emotional language was not task-relevant. Furthermore, a study by Gendron et al. found that satiation of emotion-label words disrupted emotion semantic priming, thereby obstructing the perception of facial expressions (8). Emotional faces of different intensities were used in their experiments, and after participants repeatedly read emotion-label words 30 times to induce semantic satiation, target faces were briefly presented (50 ms); then, participants were required to select from two pictures of emotional faces of different intensities the picture that best matched the target face (8). After eliminating interference from memory, the researchers found that individuals responded more slowly in the consistent satiated word and face emotion consistent condition than in the inconsistent condition (8). Combined with previous studies, changes in the semantic accessibility of emotions affect the perceptual processes of facial expressions. These findings provide evidence for the involvement of language in the construction of emotion perception and support the view of the theory of constructed emotion.

The words used in the above studies were emotion-label words, that is, words that indicate a particular emotion type or emotional response (e.g., “sadness” or “smile”). When processing emotion-label words, individuals could directly understand the emotional states expressed by the words and activate the corresponding emotions. In addition to emotion-label words, another category of words with emotional meaning (e.g., “flowers”, “death”) is commonly used in studies, so-called emotion-laden words. When processing emotion-laden words, individuals associated the words with their personal experiences, which activated the corresponding emotional experiences (27–29). Researchers focused on the semantic activation of emotion-label words and emotion-laden words and found that they both induce priming effect of emotions, but at the same time, there were differences between them (16, 30, 31). The priming effects of emotion-label words prompted individuals to recognize subsequent emotional picture stimuli faster than emotion-laden words (30, 31). The researchers further found that there were similar differences between emotion-label words and emotion-laden words primed by the second language (31). In addition, emotional valence (positive and negative) might moderate the priming effects of emotion-label words and emotion-laden words. Compared with negative emotion-laden words, the priming of negative emotion-label words significantly influenced the processing of subsequent emotion picture stimuli, whereas there was no such difference between positive emotion words (16, 31). For the semantic satiation of emotion-label words and emotion-laden words, the researchers found that the semantic satiation of emotion words resulted in a longer RT in the subsequent emotional judgment task compared with the priming condition. But there were significant differences neither between the two word types nor between positive and negative emotional valence (26). They suggested that emotional semantic satiation was strictly conditioned and that valence and word type were not sufficient to observe the semantic satiation effect (26). However, in experiments restricted to specific emotion categories (e.g., anger, disgust), researchers found that semantic satiation of emotion-label words affected the perception of subsequently presented facial expressions (8, 25). According to the theory of constructed emotion, individuals construct emotion concepts by continuously assigning specific stimuli to different emotion categories during learning and experience. As the semantic expressions of specific stimuli, emotional categories of emotion-laden words need to be discriminated with the involvement of experience. Therefore, the influence of semantic satiation of emotion-laden words on facial expression perception also deserves further research.

The basic emotion theory suggests that some of the basic, elementary emotion concepts developed in language, such as “happiness” and “anger,” are derived from the universal biological nature of humans (32). However, in a linguistic analysis of 2,474 languages, Jackson et al. found that the meaning of emotion concepts was co-constructed by our variable sociocultural environment and the common functions of emotional states related to physiological homeostasis (33). The evolved neurophysiological systems provided a universal structure for the construction of emotions, while individual experiences and cultural environments caused variations in emotion semantics (33). Jackson et al.'s findings supported the theory of constructed emotion. However, based on the debate between the theory of constructed emotion and basic emotion theory, previous studies have only manipulated the semantic satiation of emotion-label words, which was still insufficient to answer whether the role of emotion words such as “happy” and “angry” in facial expression processing indicated the involvement of emotion concepts or the role of universal biological principles. Semantic satiation of emotion-label words may only mean that processing is obstructed for specific category names and does not indicate the involvement of emotional concepts from experience and culture. However, previous studies have investigated the role of emotion concepts through behavioral results, while it is still unclear how emotional semantic satiation affects the processing of facial expressions and what role it plays in the early and later facial expression processing stages. Therefore, the neurophysiological activity of emotion concept information in facial expression perception deserves further exploration.

Therefore, our study manipulated the accessibility of emotion concepts through emotion word activation and satiation and used ERPs to investigate potential mechanisms of emotion concepts in the processing of facial expressions. Previous studies have found that the semantic activation of emotion words could affect the perception of facial expressions. Experiment 1 of the present study would further distinguish between emotion-label words and emotion-laden words to explore the effect of their emotion activation on facial expression perception. We hypothesized that if there was processing of emotional semantics in facial expression perception, priming effects of label words and laden words would affect facial expression perception and exhibit a significant consistency effect. In addition, there might be differences in emotional semantic activation between label words and laden words. Compared with laden words, the straightforward activation of emotional states of label words could facilitate the perception of subsequent emotional faces and thus exhibit shorter RTs. In Experiment 2, we examined how the semantic satiation of emotion-label words and emotion-laden words affected the perception of facial expressions. We hypothesized that satiation of label words and laden words would result in satiation of emotion semantics, which obstructed the perception of facial expressions. In terms of behavioral results, this would cause a change in the consistency effect. In terms of ERP components, if this obstruction occurred in the early stage of facial expression perception, the N170 component would exhibit a change between consistency conditions; if this obstruction occurred in the later stage, the N400 component would exhibit a change in consistency conditions, resulting in a reduced N400 effect.

## Experiment 1

Experiment 1 would explore the role of the activation of emotional concepts in language on the perception of facial expressions using emotion word priming.

### Method

#### Participants

In total, 55 college students (23 males) were recruited from different majors at Ningbo University through the Internet. The mean age was 20.45 years (*SD* = 1.31). All participants had normal or corrected-to-normal visual acuity and no color blindness or color weakness and were right-handed. The current study was approved by the Ethics Committee of Ningbo University. All participants provided written informed consent before the experiment in accordance with the Helsinki Declaration. The power analyses showed that for the current sample size, the statistical power of the main results in this study was above 0.7, and the effect size was larger than the medium effect (34, 35).

#### Stimuli

Materials for the experiment's facial expressions were chosen from the KDEF (The Karolinska Directed Emotional Faces) collection (36). The valence and arousal of six different types of facial expression pictures (happiness, sadness, anger, fear, disgust, surprise, and neutral) were rated on a 9-point scale by 30 college students (12 males and 18 females; *M*_age_ = 20.82, *SD*_age_ = 2.17) who were not involved in the formal experiment. For the valence, “1” is very negative, and “9” is very positive. For arousal, “1” is very low and “9” is very high. Based on the rating results, the main emotion types investigated in this study were happy and disgust, which had the highest and lowest valence scores, respectively. Furthermore, 10 happy faces and 10 disgusted faces with similar arousal scores were selected from the two types of faces, half of each sex. All pictures were converted to grayscale, and the external features like hair and ears were removed from the pictures, keeping only the internal features.

As word materials for the experiment, two-character Chinese emotion-label words and emotion-laden words associated with happiness and disgust were chosen. The valence and arousal of happy and disgusted emotion-label words and emotion-laden words were rated on a 9-point scale by 30 college students (14 males and 16 females; *M*_age_ = 21.37, *SD*_age_ = 1.70) who did not take part in the formal experiment. In total, 32 emotion words were used as the word materials, including eight label words and eight laden words related to happiness (e.g., joyful, flower) and eight label words and eight laden words related to disgust (e.g., dislike, maggot; see the Supplementary material for evaluation details and results).

#### Task and procedure

**Figure 1:**
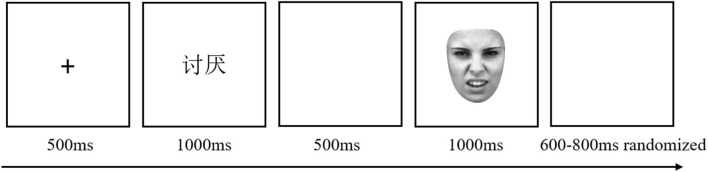
Example of a single trial. A fixation point “+” was displayed for 500 ms, then the emotion word [in emotion-label words condition, e.g., 讨厌 (tao yan), meaning disgusted in English] for 1,000 ms, a blank screen for 500 ms, and finally the face picture. By pressing buttons (F/J), participants were required to classify the type of facial expression (happy/disgusted) as quickly and accurately as possible. The assignment of buttons to expression was balanced across participants. The picture disappeared after the participant pressed the button and was presented for no more than 1,000 ms. After that, start the next trial after a random interval of 600–800 ms. Face reproduced from Lundqvist et al. (36) with permission from Karolinska Institute, licensed under a CC BY 4.0.

To make the sex ratio of the two groups as equal as possible, the participants were semi-randomly assigned to the two groups: specifically, odd-numbered male participants to the labeled word group and even-numbered male participants to the laden word group, and the opposite for females. Each group, the label words group (*N* = 28, 11 males) or the laden words group (*N* = 27, 12 males), received only the emotion-label words or emotion-laden words stimulus material. In the facial expression judgment task, the emotion word was presented for 1,000 ms [e.g., presenting “讨厌” (tao yan) in the label words group, which means disgust in English; presenting “鲜花” (xian hua) in the laden words group, which means flower in English], and then, the picture of the face was presented. By pressing buttons (F/J), participants were required to classify the type of facial expression (happy/disgusted) as quickly and accurately as possible (see Figure 1). There were 136 trials in the experiment, including 16 practice trials and 120 formal trials, 30 trials for each experimental condition.

### Results

#### Behavioral performance

For RT and ACC, trials outside the mean RTs ± 2.5 *SD* and trials with RTs longer than 2,500 ms were deleted. Deleted trials accounted for 3.6% of the total trials. Then, repeated-measures analyses of variance (ANOVAs) of 2 (word types: label words group, laden words group) × 2 (facial expressions: happy face, disgusted face) × 2 (consistency: consistent, inconsistent) were conducted for RT and ACC: word types as between-subject factors, and facial expressions and consistency as within-subject factors.

For the analysis of RT, the main effect of facial expressions was significant [*F*_(1, 53)_ = 6.06, *p* = 0.017, η^2^_*p*_ = 0.10]; the happy face (*M* = 671 ms, *SE* = 19.72) was faster than the disgusted face (*M* = 682 ms, *SE* = 20.71). Additionally, there was a significant interaction effect between word types and facial expressions [*F*_(1, 53)_ = 6.21, *p* = 0.016, η^2^_*p*_ = 0.11]. Further analysis revealed that the happy face was significantly faster than the disgusted face for the label words group (happy face: *M* = 669 ms, *SE* = 27.64; disgusted face: *M* = 691 ms, *SE* = 29.02, *p* = 0.001); for the laden words group, there was no significant difference across facial expressions (happy face: *M* = 673 ms, *SE* = 28.14; disgusted face: *M* = 672 ms, *SE* = 29.55, *p* = 0.984). The main effect of consistency was also significant [*F*_(1, 53)_ = 19.48, *p* < 0.001, η^2^_*p*_ = 0.27], indicating that the consistent condition (*M* = 659 ms, *SE* = 17.49) was significantly faster than the inconsistent condition (*M* = 694 ms, *SE* = 23.11).

**Figure 2:**
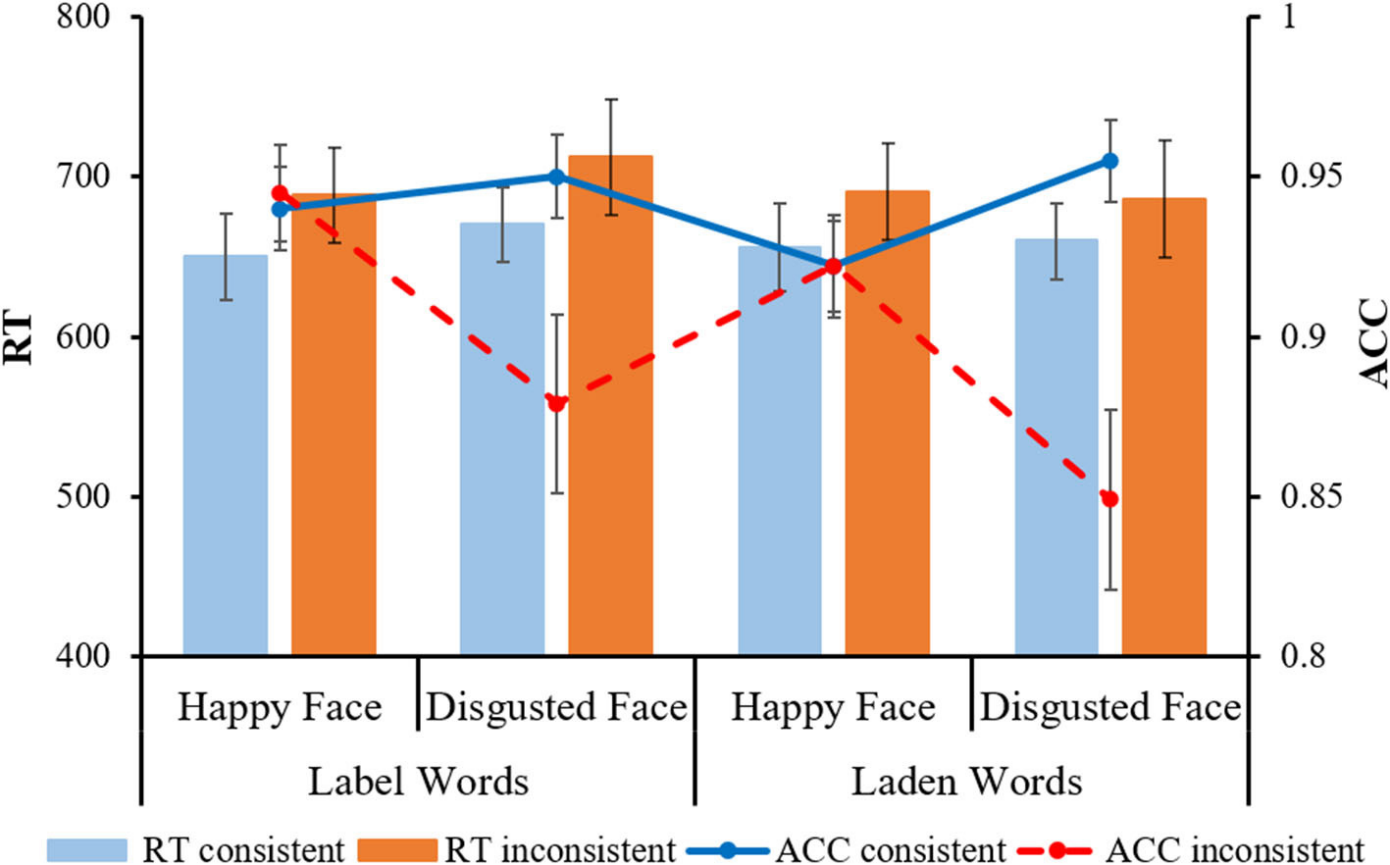
Participants' reaction time and accuracy in different conditions of the facial expression judgment task. The error line indicates the standard error (*SE*).

For the analysis of ACC, the main effect of facial expressions was significant [*F*_(1, 53)_ = 7.15, *p* = 0.01, η^2^_*p*_ = 0.12], indicating that the happy face (*M* = 0.93, *SE* = 0.01) was more accurately classified than the disgusted face (*M* = 0.91, *SE* = 0.01). The main effect of consistency was also significant [*F*_(1, 53)_ =13.55, *p* = 0.001, η^2^_*p*_ = 0.20], indicating that the consistent condition (*M* = 0.94, *SE* = 0.01) was higher than the inconsistent condition (*M* = 0.90, *SE* = 0.01). In addition, there was a significant interaction effect between consistency and facial expressions [*F*_(1, 53)_ = 19.58, *p* < 0.001, η^2^_*p*_ = 0.27]. Further analysis indicated that the consistency condition had a significantly higher ACC than the inconsistent condition for the disgusted face (consistent: *M* = 0.95, *SE* = 0.01; inconsistent: *M* = 0.86, *SE* = 0.02, *p* < 0.001), while for the happy face, the difference in ACC between consistent and inconsistent was not significant (consistent: *M* = 0.93, *SE* = 0.01; inconsistent: *M* = 0.93, *SE* = 0.01, *p* = 0.819) (see Figure 2).

### Discussion

Experiment 1 investigated the priming effect of emotion-label words and emotion-laden words on facial expression recognition. The results showed that facial expression recognition was influenced by emotion words. Both the RT and ACC of facial expression recognition showed significant main effects of consistency. This is in line with many previous studies on the language context effect (7, 37). More specifically, the happy face was more easily recognized and had better performance on RT and ACC, while the disgusted face was more susceptible to emotion words and thus had reduced ACC in inconsistent conditions. These suggested that there might be differences in the processing of happy and disgusted facial expressions. In terms of word types, there were no significant differences between the label group and the laden group, but the interaction effect between word types and facial expressions was found: There was a difference in RT between happy and disgusted faces in the label group but not in the laden group. These results were similar to those of a previous study (31) and partially supported the hypothesis that there were differences between label words and laden words in terms of emotional activation. We suggested that although both types of words could induce corresponding emotional states and thus exhibit the significant main effect of consistency, label words could be processed more efficiently, which saved cognitive resources and thus allowed the dominance of happy faces to be presented. In contrast, the processing of laden words consumed more cognitive resources, which might mask the minor differences in the subsequent processing of happy and disgusted faces.

## Experiment 2

Experiment 2 would further explore the obstructing effect of semantic satiation of emotion words to verify, from another perspective, the role of the emotion concept externalized in language in the perception of facial expressions.

### Method

#### Participants

In total, 45 college students (13 males) were recruited from different majors at Ningbo University through the Internet. The mean age was 20.16 years (*SD* = 1.08). The requirements for the participants were the same as for Experiment 1. The data of three participants were eliminated because they had more artifacts than 20% of the total number of epochs in the ERP analysis, and the data of the remaining 42 participants were used for statistical analysis. The power analyses showed that for the current sample size, the statistical power of the main results in this study was above 0.8, which was in line with Cohen's regarding the statistical power of psychological study (34, 35).

#### Stimuli

The face and word materials used in the experiment were the same as those used in Experiment 1. We additionally recruited 30 participants who did not participate in the formal experiment to rate neutral words (such as steel, train), and then, based on the ratings, chose eight neutral words as filler stimulus materials to be used in the experiment (see the Supplementary material for evaluation details and results).

#### Task and procedure

**Figure 3:**
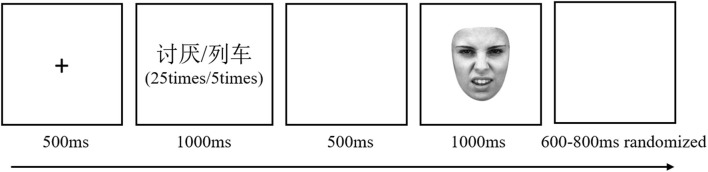
Example of a single trial. A fixation point “+” was displayed for 500 ms, followed by 30 times emotion word [in the emotion-label words group, e.g., 讨厌 (tao yan), meaning disgusted in English] or neutral word [e.g., 列车 (lie che), meaning train in English]. When the word was presented, the participants were required to read the word silently and determine whether the word had emotional meaning: if related, no key was pressed, and if not, the space bar was pressed. Then, an empty screen was presented, followed by a face picture. By pressing buttons (F/J), participants had to determine the type of facial expression (happy/disgusted), and the order of buttons was balanced between the participants. After that, start the next trial after a random interval of 600–800 ms. Face reproduced from Lundqvist et al. (36) with permission from Karolinska Institute, licensed under a CC BY 4.0.

Participants were randomly assigned to one of two groups: label words group (*N* = 22, seven males) or laden words group (*N* = 23, six males). After the presentation of the “+,” the words were presented 30 times in the center of the screen (in the experiment trials, the same emotion word was repeated 25 times and the neutral word was repeated five times, and the order of presentation of words was pseudo-random: The neutral word was presented only five times within the first 15 times, and the emotion word was repeatedly presented more than 15 times in the remaining times, thus achieving semantic satiation; in the filler trials, the neutral word was repeated 25 times and the emotion-label word was repeated five times, and the order of presentation of words was random). When the word was presented, the participants were required to read it silently and determine whether it had emotional meaning. Then an empty screen was presented, followed by a facial expression type judgment task in which participants were required to make a quick judgment of the expression type (see Figure 3). There were 180 trials in the experiment, including four practice trials, 160 experiment trials, 16 filler trials, and 40 trials for each experimental condition. In the experiments, neutral words and filler trials served to mask the purpose of the experiment and were not included in the data analysis.

#### Data recording and ERP analysis

The electroencephalogram (EEG) data were recorded and analyzed using the Neuroscan SynAmps2. A 68 Ag/AgCl electrode cap was used to record EEG data at the scalp position as well as vertical electrooculogram (VEOG) and horizontal electrooculogram (HEOG), with the electrode positioned according to the international 10–20 system. The reference electrode was an external electrode placed on the left ear mastoid. The HEOG recording electrodes were placed at 10 mm on the outer side of the lateral canthi of both eyes, and the VEOG recording electrodes were placed at 10 mm above and below the left eye. The sampling rate was 500 Hz. All electrodes' impedances were kept below 5 kΩ.

The EEGLAB toolbox (38) was used to analyze the EEG data. Data were bandpass filtered from 0.5 to 30 Hz and segmented into epochs from 100 ms pre-stimulus to 800 ms post-stimulus. The epochs were stimulus-locked to face-onset, and the first 100 ms was the baseline. After manually excluding epochs with larger drift, independent component analysis (ICA) was used. Visual identification and rejection of a number of independent components that represented artifacts like ocular movement. After removing the epochs with peak-to-peak deflection values higher than ±100 μV, each type of sequence was averaged separately. Based on previous research and combined with collapsed localizers of our average data, time windows and regions of interest (ROIs) were selected (39). For N170, we found that the wave peaks around 190 ms in the temporo-occipital region, so the left (P7, PO7) and right (P8, PO8) temporo-occipital electrode clusters for the 155–205 ms time window were selected. For N400, we found that both the frontocentral and centroparietal regions showed negative-going deflection and peaks around 330 ms, so the frontocentral (F1, Fz, F2, FC1, FCz, FC2) and centroparietal (CP1, CPz, CP2, P1, Pz, P2) electrode clusters for the 290–420 ms time window were selected. Mean amplitudes were analyzed by ANOVAs with repeated measurements.

### Results

#### Behavioral performance

**Figure 4:**
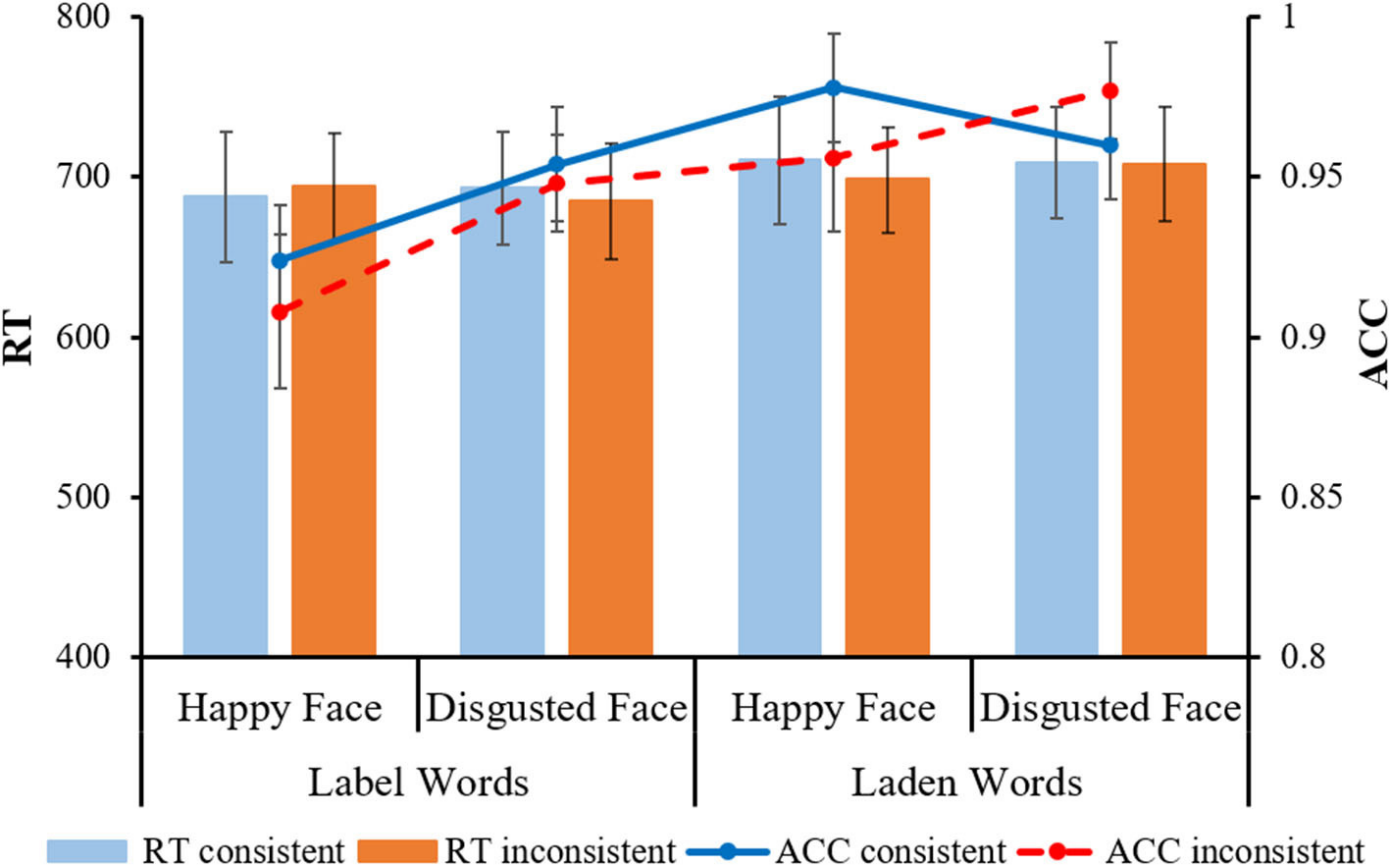
Participants' reaction time and accuracy in different conditions of the facial expression judgment task. The error line indicates the standard error (*SE*).

Behavioral data were filtered using the same method as in Experiment 1. Deleted trials accounted for 1.3% of the total trials. Repeated-measures ANOVAs of 2 (word types: label words group, laden words group) × 2 (facial expressions: happy face, disgusted face) × 2 (consistency: consistent, inconsistent) were analyzed for RT and ACC. The results showed that the main effects and interactions were not significant in each condition (*ps* > 0.05; see Figure 4).

#### ERPs

**Figure 5:**
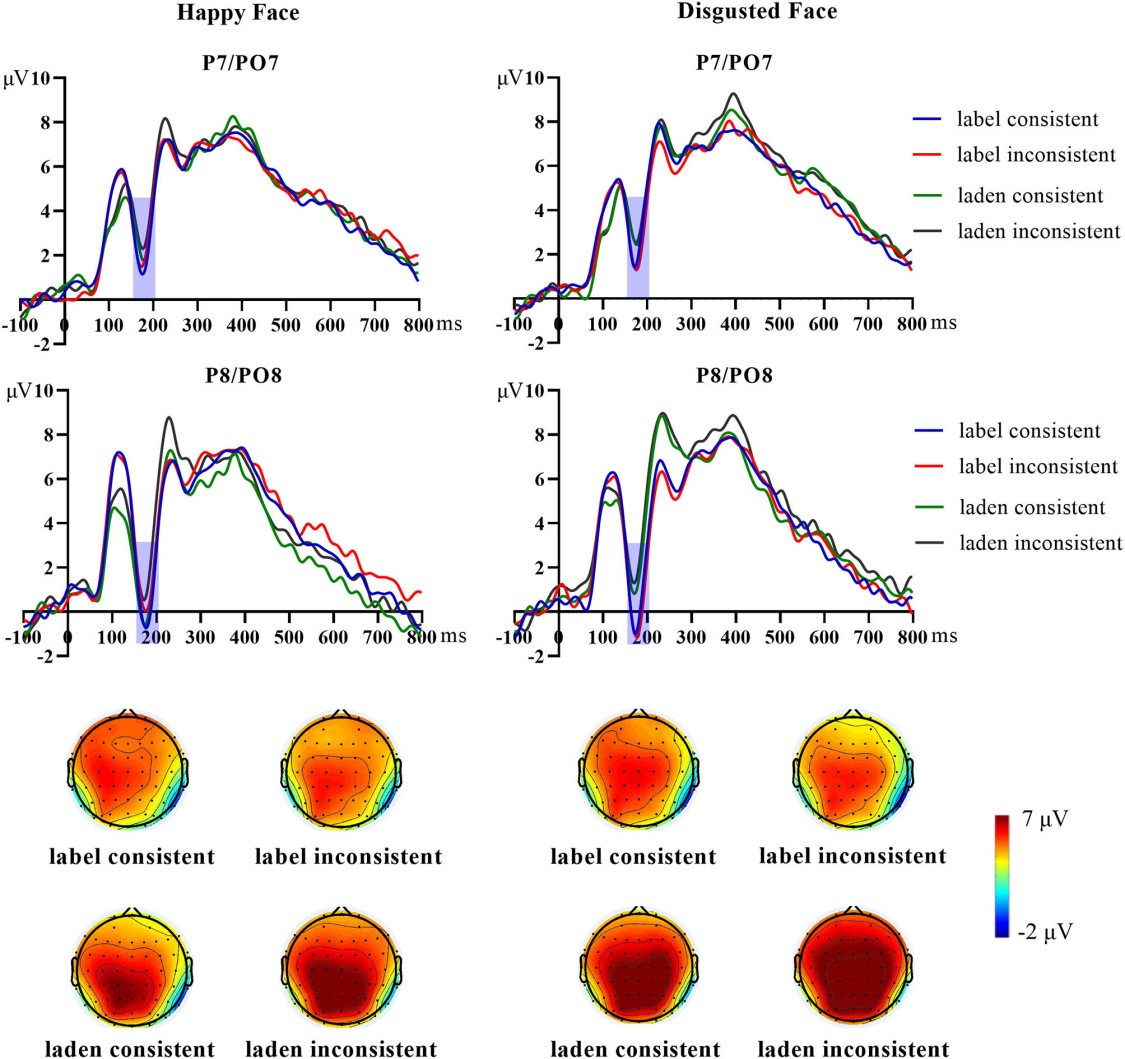
The grand average N170 elicited by happy and disgusted faces at the ROI (left hemisphere: P7/PO7, right hemisphere: P8/PO8) and the scalp topography of each condition.

**Figure 6:**
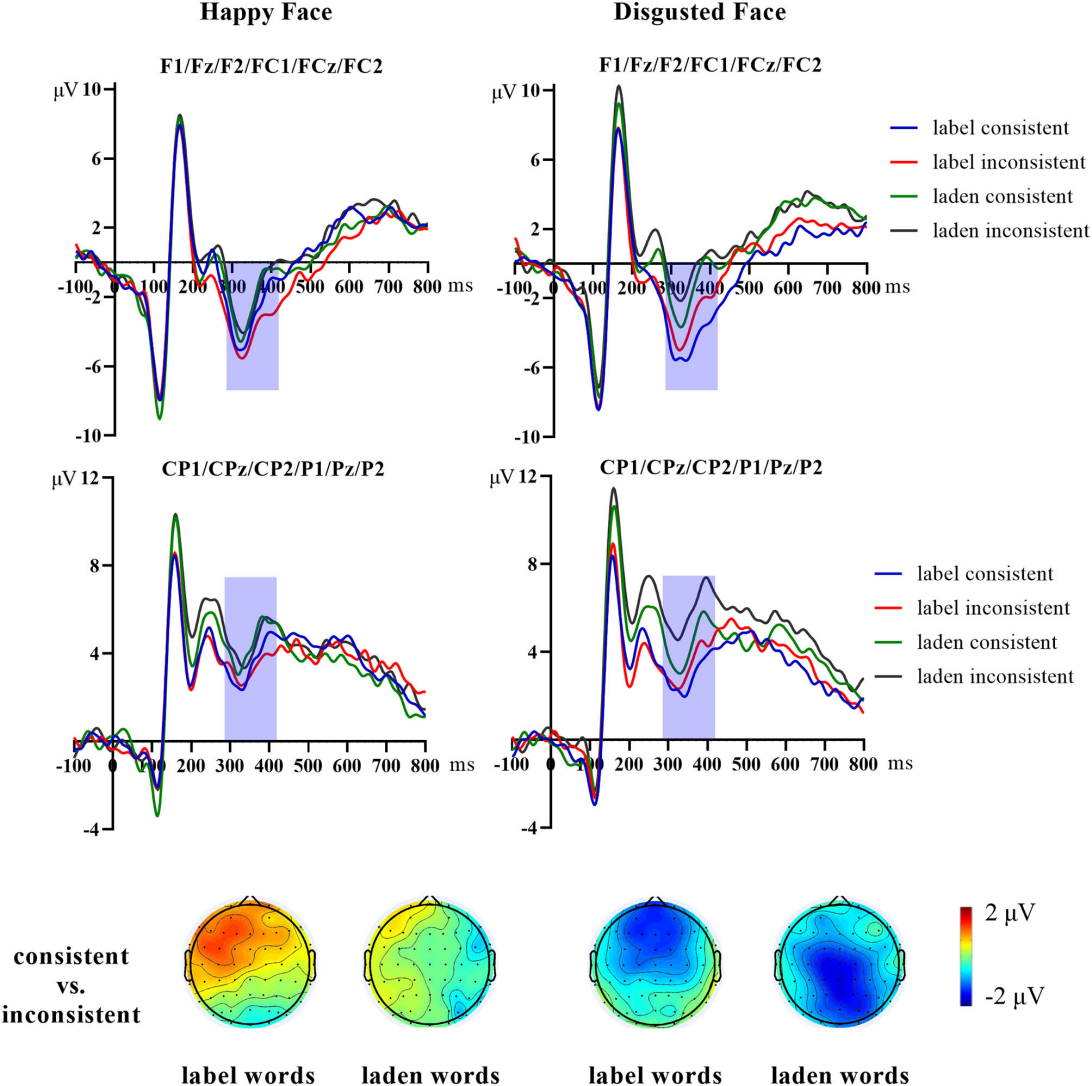
The grand average N400 effect elicited by happy and disgusted faces at the ROI (frontocentral: F1/Fz/F2/FC1/FCz/FC2; centroparietal: CP1/CPz/CP2/P1/Pz/P2). The consistent condition minus the inconsistent condition was used to calculate the scalp topographies.

Figure 5 shows the scalp topographies and grand average of N170 elicited by happy and disgusted facial expressions. Figure 6 shows the scalp topographies and grand average N400 effect elicited by happy and disgusted facial expressions.

##### N170 components

The mean amplitude of N170 was analyzed by repeated-measures ANOVAs of 2 (word types: label words group, laden words group) × 2 (facial expressions: happy face, disgusted face) × 2 (consistency: consistent, inconsistent) × 2 (hemispheres: left hemisphere, right hemisphere). The results showed that the main effect of hemispheres was significant [*F*_(1, 40)_ = 6.83, *p* = 0.013, η^2^_*p*_ = 0.15]. The hemispheres and consistency had a significant interaction effect [*F*_(1, 40)_ = 5.59, *p* = 0.023, η^2^_*p*_ = 0.12]. Further analysis indicated that the N170 amplitude for the consistent condition was more negative than the inconsistent condition in the right hemisphere (consistent: *M* = 1.16 μV, *SE* = 0.80; inconsistent: *M* = 1.70 μV, *SE* = 0.73, *p* = 0.028); while in the left hemisphere, the difference was not significant (consistent: *M* = 2.91 μV, *SE* = 0.59; inconsistent: *M* = 3.07 μV, *SE* = 0.58, *p* = 0.40). In addition, there was a significant interaction effect between word types and facial expressions [*F*_(1, 40)_ = 5.93, *p* = 0.019, η^2^_*p*_ = 0.13], and there was a significant interaction effect between the hemispheres, word types, and facial expressions [*F*_(1, 40)_ = 13.29, *p* = 0.001, η^2^_*p*_ = 0.25]. Further analysis indicated that for the laden words group, the N170 amplitude of the happy face was more negative than the disgusted face in the right hemisphere (happy face: *M* = 1.55 μV, *SE* = 1.02; disgusted face: *M* = 2.66 μV, *SE* = 1.09, *p* < 0.001). The differences between all other conditions were not significant (*p*s > 0.05).

##### N400 effect

The mean amplitude of N400 was analyzed by repeated-measures ANOVAs of 2 (word types: label words group, laden words group) × 2 (facial expressions: happy face, disgusted face) × 2 (consistency: consistent, inconsistent) × 2 (ROIs: frontocentral, centroparietal). The results showed that the main effect of ROIs was significant [*F*_(1, 40)_ = 204.19, *p* < 0.001, η^2^_*p*_ = 0.836]. In addition, there was a significant interaction effect between consistency and facial expressions [*F*_(1, 40)_ = 5.27, *p* = 0.027, η^2^_*p*_ = 0.12]. More importantly, there was a significant interaction effect between the word types, consistency, ROIs, and facial expressions [*F*_(1, 40)_ = 7.71, *p* = 0.008, η^2^_*p*_ = 0.16]. Further analysis indicated that for the emotion-label words group, the N400 amplitude of the disgusted faces at the frontocentral region was more negative in the consistent condition than that in the inconsistent condition (consistent: *M* = −4.43 μV, *SE* = 1.33; inconsistent: *M* = −3.14 μV, *SE* = 1.25, *p* = 0.049), while for the emotion-laden words group, the N400 amplitude of the disgusted faces at the centroparietal region was more negative in the consistent condition than it was in the inconsistent condition (consistent: *M* = 4.374 μV, *SE* = 1.29; inconsistent: *M* =5.869 μV, *SE* = 1.09, *p* = 0.024). For the happy face, there was no significant difference across conditions (*p* > 0.05).

### Discussion

In Experiment 2, we investigated the effect of semantic satiation of emotion words on the perception of facial expressions. In contrast to the emotional activation condition of Experiment 1, Experiment 2 obstructed the corresponding emotional perception by manipulating semantic satiation. In such experimental manipulation, we expected to observe a different or even reversed performance than in Experiment 1 in terms of behavioral results, thus verifying the manipulation effectivity of obstruction. It was found that the consistency effect exhibited in Experiment 1 was not significant in Experiment 2 on both RT and ACC, suggesting that semantic satiation effectively obstructed emotion perception, resulting in a different performance compared to emotion activation. There were also no differences between word types, suggesting that emotion concepts in words (not just emotional category words) were involved in facial expression processing. Unlike Lindquist et al., who found that emotion word satiation led to longer RT in the consistency condition (25), this reversal did not occur in the present study. We suggested that this variation might be due to the experimental manipulation: Only emotion-label words were used in Lindquist et al.'s study, and participants were tasked with making word-face emotion consistency judgments, which brought the role of emotional semantics into sharper focus. Nevertheless, by comparing Experiment 1 and Experiment 2, we were still able to find that the change in emotional semantic accessibility affected the perception of facial expressions.

In previous experiments of emotion priming, the N170 component amplitude was larger in the inconsistent condition than in the consistent condition (13, 15). In contrast, a consistency effect with opposite performance was shown in the present study, that is, the consistent condition was larger than the inconsistent condition. The obstructive effect of semantic satiation on the N170 component suggested that the extraction of emotional information about the faces already existed in the early stage of facial expression processing. The difference in word types might imply that semantic satiation of labeled words had a stronger obstructive effect, thus masking the difference between happy and disgusted faces, while happy faces still exhibited processing dominance in the laden words semantic satiation condition. At the later stage, compared with the inconsistent condition, disgusted faces showed a more negative N400 amplitude in the consistent condition, exhibiting the reversed N400 effect compared with previous semantic activation studies (13, 18, 19). This suggested that the semantic satiation of disgusted emotion words was transferred to the emotional perception of the consistent condition and made it hard to categorize and that the semantic satiation of emotion-label words and emotion-laden words had the same effect. Faces in the inconsistent condition were not affected by semantic satiation and so elicited a relatively reduced N400 amplitude. In addition, we found differences in the distribution of N400 effects between word types. The emotion-label words condition exhibited a more frontally distributed N400 (i.e., an FN400), while the emotion-laden words condition exhibited a parietally distributed, more conventional N400. Voss and Federmeier suggested that FN400 and N400 have the same function and both reflect cognitive conflict due to semantic inconsistency (40). However, Bridger et al. and Strozak et al. separated semantic priming test and semantic recognition test and found that FN400 was also associated with semantic information familiarity as well as the recognition process (41, 42). However, in Hoemann et al.'s study exploring the acquisition of emotional concepts, the FN400 was considered to have similar functions to the N400 that reflect the processing of emotional meaning (21). The functional differences between N400 and FN400 in semantic research are still debated by researchers. Whether this difference in the topographical distribution in this study reflects word type differences or different cognitive processes in face perception remains to be further explored. Combining the results of Experiment 1, we found that disgusted faces were more dependent on emotional concepts from language and had different performances in the emotion semantic activation and satiation conditions.

## General discussion

Based on the controversies of basic emotion theory and constructed emotion theory, our study examined the influence of emotion-label words, emotion-laden words activation, and satiation in the perception of facial expressions in order to investigate whether the perception of facial expressions was based on specific neural mechanisms of face structure or was influenced by top-down emotion concepts.

The constructed emotion theory states that emotional concepts derived from learning and experience are involved in facial expression perception. Our results provided evidence for this view. In terms of behavioral data, the present research showed similar results to previous studies on the language context effect (7, 43, 44), that is, promotion in the consistent condition, which suggested that facial expression perception is not only based on structural features of faces. Furthermore, in the semantic satiation condition, facial expression perception no longer exhibited the consistency effect and even showed the reverse result for the ERP components compared with the semantic activation condition, and the consistent condition always elicited more negative ERP components in the early and later stages. The current view is that N400 reflects the processing and integration of meaningful stimuli (20). In a word-face emotion consistency judgment task, Krombholz et al. used the emergence of the N400 effect as an index of emotional semantic processing (45). By examining the difference waves of the inconsistent minus consistent condition, they discovered that the word-face emotion inconsistent condition exhibited a significant N400 effect (45). Li et al. also found that disgusted faces elicited more negative N400 amplitudes in the happy word priming condition than in the disgusted word priming condition (16). Thus, the researchers believed that there was activation of emotional semantics in face emotion processing. The present study further found that the N400 amplitude of disgusted faces in the consistent condition was significantly more negative than that in the inconsistent condition when there was semantic satiation of emotion words, showing a reversed N400 effect, which means that emotional semantic satiation obstructs the perception of the same type of emotional faces and even produces cognitive conflict. These results suggested that the perception of facial emotions is linked to language and the emotional category to which it belongs. Language, however, is located in the cultural context of the individual and is the framework of face emotion categorization and the externalization of emotional concepts (6, 8). The results of our study were also supported by cognitive neuroscience. Lindquist et al. performed a meta-analysis of fMRI data related to emotion perception and found that in addition to core brain regions associated with emotions (e.g., regions of the hippocampus, amygdala, and cingulate gyrus) being activated during emotion generation and emotion recognition, the anterior temporal cortex and left prefrontal cortex associated with language processing were also activated (46). This suggested that emotion perception was an integral component of the overall cognitive network rather than a basic mental process generated by a separate neural mechanism. Thus, the construction of the emotion concept system also influences how individuals perceive emotions through facial expressions.

Emotion words are produced in communication and are influenced by the context of society and culture. It has been found that emotion-label words and emotion-laden words evoke different neural activity in the cerebral cortex (27, 47, 48). However, we found that they both activated emotions as priming stimuli and obstructed the perception of facial expressions after semantic satiation. The same performances were also observed for the ERP components. Neely introduced the spreading activation model to the study of semantic satiation (49). He proposed that semantic satiation of the priming words would inhibit the spread of activation between semantically related morphemes in the semantic network or inhibit individuals' attention to semantically related morphemes, thus resulting in difficulties in semantic activation (49). Smith and Klein found that after multiple repetitions of the word at the upper node of the semantic network (e.g., “fruit”) until semantic satiation occurred, individuals were slower to judge words belonging to that category and located at its lower node (e.g., “apple”), even though the word “apple” was not semantically satiated (50). In our Experiment 2, the semantic satiation of emotion-label words that directly provided information about the emotion category affected the perception of the same type of emotional faces, which conformed to the spreading activation model in which the satiation of the upper node inhibited the lower node of the same category. The emotion-laden words were actually in the lower nodes of the emotion semantic network, but after the semantic satiation manipulation, they also resulted in the satiation of the emotion category in the upper nodes and inhibited the processing of the same category of emotion faces, which is the same as the satiation of emotion-label words. The results suggested that the perception of facial expressions relied on the activation of emotion concepts and might share a common emotional processing pathway with emotion words. This emotional semantic satiation might be limited to a specific emotional category since the satiation effect was hardly observed by satiating only positive and negative valence words (26). Furthermore, satiation of emotion-laden words at the lower node might propagate backward along the emotional semantic network, leading to the satiation of the emotion category in the upper node and obstructing facial expression perception.

The present study found that there were relatively stable differences in individuals' perceptions of happy and disgusted emotions. Disgusted face processing was more susceptible to emotion words and exhibited a reversed N400 effect in both emotion-label word and emotion-laden word semantic satiation experiments. In contrast, happy faces showed consistency effects only in the semantic priming experiment, and no significant differences in N400 amplitudes were observed in the semantic satiation experiments. Previous studies exploring the semantic satiation of emotions have ignored the differences in emotion valence, especially the different representations between positive and negative emotions. For example, Lindquist et al. and Gendron et al. only discussed differences in word activation, satiation conditions, and face emotion consistency without separating specific emotion types (8, 25). Gendron et al. used a series of negative emotion faces and found that when the word “disgust” was semantic satiation, it obstructed the perception of even angry faces in addition to disgusted faces (8). They suggested that negative emotion perception was consistent with the spreading activation model hypothesis: activation and satiation of a negative emotion type spread through the semantic network, which in turn affected the perception of other negative emotion types. However, happy emotion might be at the other pole of the emotional valence dimension compared to other negative emotions, so the results of studies on negative emotion perceptions made it difficult to reveal the processing pattern of happy emotion. Previous studies have found that individuals performed better in detecting and recognizing happy faces, and there was a happy face dominance effect (51–53). In a change detection task, Švegar and Kardum compared the RTs and ACC rates of seven basic facial expression pictures (52). They found that changes in happy faces were detected more quickly and accurately than changes in other emotional types of faces. Becker et al. explored the differences between different facial expressions in a visual search task after excluding possible confounding by physical features (53). They found that individuals showed dominance for happy face detection in both single emotional face detection and multiple emotional face detection tasks. Researchers suggested that the emotional meaning of happy faces was clearer and more conducive to interpersonal communication than other confusing negative emotional faces, and therefore, the happy face dominance effect might stem from a specific processing mechanism. In the present study, individuals' processing of happy faces was less affected by emotional words in semantic satiation experiments, which also suggested that the processing of happy faces might be more dependent on the visual encoding of facial configuration features.

The present study still has some limitations. Through the pre-experiment, we selected the two types of emotional faces with the highest valence scores, and future studies would select more emotion categories, especially negative emotions such as fear, anger, and sadness, to clarify whether there is a stable effect of emotional words in the perception of negative emotions. In addition, emotion-laden words were more dependent on cultural environment and life experiences, and the understanding of the emotional meaning of words might vary among individuals. Although the present study selected word materials through a pre-experiment, future studies still need to pay attention to the issue of individual differences. Finally, sex differences in facial expression perception were also of concern. It has been found that male participants showed a larger happy face facilitation effect of male faces compared to female faces in expression recognition (54). Female participants were found to have an advantage in the recognition of negative emotions, such as fear and anger (55). Moreover, sex differences in facial expression perception were also influenced by other factors (56). Future research needs to further balance the sex ratio of participants and focus on the sex differences in emotion word processing and its impact on facial expression perception.

## Conclusion

(1) Emotion concept information influenced the perception of facial expressions, but there were differences between happy and disgusted faces. Disgusted faces were more dependent on emotion concept information and showed different performances in semantic activation and satiation conditions.

(2) Emotion-label words and emotion-laden words both obstructed the perception of disgusted facial expressions after semantic satiation and induced a more negative N400 in the emotion consistency condition, showing a reversed N400 effect.

(3) Facial expressions would be processed with the involvement of emotion concepts in the form of language, which included a broader range of emotional words, including emotion-laden words and not limited to emotion-label words.

## Data availability statement

The raw data supporting the conclusions of this article will be made available by the authors, without undue reservation.

## Ethics statement

The studies involving human participants were reviewed and approved by Ethics Committee of Ningbo University. The patients/participants provided their written informed consent to participate in this study.

## Author contributions

QX: conceptualization. WW: data curation. YY: methodology. WL: writing—original draft. All authors contributed to the article and approved the submitted version.
