# Supplementary material for: Effects of emotion words activation and satiation on facial expression perception: evidence from behavioral and ERP investigations
Source: Front Psychiatry. 2023 Jul 31;14:1192450. doi: 10.3389/fpsyt.2023.1192450 (PMC10425554; doi:10.3389/fpsyt.2023.1192450)
Supplement: Supplementary file 1 [file Data_Sheet_1.DOCX]

**Supplementary Material**

**Faces and words material evaluation experiments**

The facial expression materials used in the experiment were selected from the KDEF set (The Karolinska Directed Emotional Faces; Lundqvist, Flykt & Öhman, 1998). 30 college students who did not participate in the formal experiment were recruited via the Internet to rate the valence and arousal of six types of facial expression picture (happiness, fear, sadness, disgust, anger, surprise, and neutral) on a 9-point scale. For the valence:1 was very negative, 9 was very positive. For the arousal: 1 was very low, 9 was very high. The results showed that, in terms of emotional valence, happy faces were rated the most positive valence scores and disgusted faces were rated the most negative valence scores, which were significantly different from other emotion types (*ps* < 0.001). In terms of arousal scores, there was no significant difference between happy faces and disgusted faces (*p* = 0.598). Detailed results are shown in **Table 1**.Therefore, happiness and disgust were selected as the emotion types discussed in this study. 10 happy faces and 10 disgusted faces with similar arousal scores were selected, half men and half women. Repeated measurement ANOVA analysis of the selected faces found that there were significant differences in valence scores between happy and disgusted faces, *F* (1, 29) = 361.45, *p* < 0.001, η^2^*_p_* = 0.92; the score of happy faces (7.28±0.72) was significantly larger than disgusted faces (2.86±0.7). In terms of arousal score, *F* (1, 29) = 0.091, *p* = 0.765, η^2^*_p_* = 0.003, there was no significant difference between happy faces (6.21±1.24) and disgusted faces (6.47±1.20).

Table1. Valence and arousal scores of six types of emotional face picture(*M±SE*)

| Emotional type | Valence | Arousal |
| --- | --- | --- |
| disgusted | 2.43±0.88 | 5.01±1.48 |
| anger | 2.81±0.72 | 4.74±1.25 |
| sadness | 2.86±0.73 | 4.40±1.24 |
| fear | 3.15±0.77 | 4.84±1.08 |
| surprise | 4.48±0.94 | 4.77±1.20 |
| neutral | 4.60±0.50 | 3.45±1.30 |
| happy | 6.89±0.71 | 5.76±1.11 |

Two-character Chinese emotion-label words and emotion-laden words related to happy and disgusted, as well as neural words were selected as word materials used in the experiment. 30 college students who did not participate in the formal experiment were recruited via the Internet to rate the valence and arousal of happy, disgusted emotion-label words and emotion-laden words on a 9-point scale. We also made a comprehensive comparison of word superiority, familiarity, word frequency, phrase frequency, stroke numbers, etc. 8 words of each type were selected after evaluation. The valence and arousal scores of the selected words were analyzed by repeated measurement ANOVA of 2(word types: emotion label word, emotion laden word) ×2(emotion types: happy, disgusted).In terms of valence score, the main effect of emotion types was significant (*F* (1, 7) = 2775.69, *p* < 0.001, η^2^*_p_* = 0.99). The score of happy words (7.21±0.34) was significantly larger than of disgusted words (2.61±0.32), and there was no significant difference between label and laden words (*F* (1, 7) = 1.39, *p* = 0.276, η^2^*_p_* = 0.16). In terms of arousal score, the main effects of emotion types and word types and their interaction effect were not significant, and there was no significant difference between happy words (5.36±0.33) and disgusted words (5.16±0.35).

Table2. Rating results for happy emotion-label words and emotion-laden words.

|  | Happy words | Valence | *SD* | Arousal | *SD* | Dominance | *SD* | familiarity | *SD* | concreteness | *SD* | First word  stroke number | Second word stroke number | lexical frequency |
| --- | --- | --- | --- | --- | --- | --- | --- | --- | --- | --- | --- | --- | --- | --- |
| Label words  (All mean happiness) | 快乐 | 7.83 | 1.22 | 5.72 | 1.93 | 5.92 | 1.83 | 6.53 | 1.72 | 2.47 | 0.62 | 7 | 5 | 0.0037 |
|  | 高兴 | 7.74 | 1.03 | 5.23 | 1.85 | 6.01 | 1.72 | 6.72 | 1.82 | 3.21 | 2.03 | 10 | 6 | 0.0176 |
|  | 开心 | 7.25 | 0.98 | 4.98 | 2.06 | 6.54 | 1.66 | 6.23 | 1.78 | 3.03 | 1.74 | 4 | 4 | 0.0011 |
|  | 愉快 | 7.07 | 1.28 | 5.33 | 2.12 | 6.54 | 1.63 | 6.38 | 1.81 | 2.47 | 1.84 | 12 | 7 | 0.0048 |
|  | 欢乐 | 7.24 | 1.11 | 5.40 | 2.09 | 6.47 | 1.72 | 6.53 | 1.67 | 3.67 | 2.30 | 6 | 5 | 0.0029 |
|  | 欢喜 | 7.12 | 1.52 | 5.25 | 1.95 | 6.47 | 1.78 | 6.21 | 1.89 | 2.70 | 0.98 | 6 | 12 | 0.0023 |
|  | 喜悦 | 7.33 | 1.35 | 5.22 | 1.97 | 6.23 | 1.87 | 6.01 | 1.63 | 3.48 | 1.23 | 12 | 10 | 0.0293 |
|  | 愉悦 | 6.92 | 1.27 | 5.06 | 2.02 | 6.01 | 1.53 | 6.22 | 1.75 | 2.92 | 1.08 | 12 | 10 | 0.0048 |
| Laden words | 喜剧(comedy) | 7.01 | 1.17 | 5.30 | 2.04 | 6.05 | 1.91 | 5.64 | 1.97 | 2.50 | 1.64 | 12 | 10 | 0.0014 |
|  | 祝福(wishes) | 7.59 | 0.93 | 5.17 | 1.89 | 6.80 | 1.59 | 6.61 | 1.61 | 3.21 | 2.35 | 9 | 13 | 0.0009 |
|  | 友谊(friendship) | 7.49 | 1.12 | 5.58 | 2.02 | 6.32 | 1.92 | 6.70 | 1.70 | 3.05 | 2.70 | 4 | 10 | 0.0031 |
|  | 庆祝(celebration) | 6.89 | 1.33 | 5.69 | 2.22 | 6.20 | 1.68 | 6.07 | 1.91 | 2.55 | 1.82 | 6 | 9 | 0.0018 |
|  | 鲜花(flower) | 7.37 | 1.13 | 4.54 | 2.18 | 6.75 | 1.86 | 6.51 | 1.74 | 1.30 | 0.66 | 14 | 7 | 0.0017 |
|  | 漂亮(beauty) | 7.31 | 1.13 | 5.80 | 2.02 | 5.41 | 2.10 | 6.78 | 1.50 | 2.75 | 2.05 | 14 | 9 | 0.0055 |
|  | 晚会(party) | 6.53 | 1.13 | 5.82 | 1.85 | 5.72 | 1.97 | 6.34 | 1.74 | 2.60 | 1.88 | 11 | 6 | 0.0011 |
|  | 团聚(reunion) | 6.78 | 1.51 | 5.74 | 2.19 | 6.01 | 1.73 | 6.40 | 1.98 | 2.74 | 1.82 | 6 | 14 | 0.0006 |

Table3. Rating results for disgusted emotion-label words and emotion-laden words.

|  | Disgusted words | Valence | *SD* | Arousal | *SD* | Dominance | *SD* | familiarity | *SD* | concreteness | *SD* | First word  stroke number | Second word stroke number | lexical frequency |
| --- | --- | --- | --- | --- | --- | --- | --- | --- | --- | --- | --- | --- | --- | --- |
| Label words  (All mean disgusted) | 厌恶 | 2.96 | 1.26 | 5.17 | 2.23 | 5.28 | 1.99 | 5.57 | 2.03 | 3.42 | 2.19 | 6 | 10 | 0.0015 |
|  | 嫌恶 | 2.91 | 1.99 | 5.02 | 2.78 | 6.97 | 1.77 | 6.96 | 2.62 | 3.34 | 2.02 | 13 | 10 | 0.0006 |
|  | 反感 | 2.66 | 1.66 | 5.72 | 1.92 | 6.00 | 1.55 | 6.83 | 2.22 | 2.67 | 2.08 | 4 | 13 | 0.001 |
|  | 作呕 | 2.30 | 1.39 | 5.25 | 2.34 | 6.78 | 1.88 | 6.27 | 2.87 | 2.59 | 2.42 | 7 | 7 | 0.0006 |
|  | 憎恶 | 2.71 | 1.75 | 4.54 | 2.59 | 6.58 | 1.12 | 5.67 | 2.17 | 3.51 | 2.62 | 15 | 10 | 0.0006 |
|  | 讨厌 | 2.95 | 1.89 | 4.95 | 2.56 | 6.40 | 1.49 | 6.14 | 1.86 | 3.78 | 2.24 | 5 | 6 | 0.0022 |
|  | 恶心 | 2.26 | 1.89 | 5.26 | 2.96 | 6.45 | 1.10 | 6.15 | 2.59 | 3.09 | 1.91 | 10 | 4 | 0.0008 |
|  | 厌烦 | 2.25 | 1.19 | 5.30 | 2.03 | 5.80 | 1.78 | 5.42 | 2.40 | 2.72 | 2.07 | 6 | 10 | 0.0006 |
| Laden words | 苍蝇(fly) | 2.57 | 1.47 | 4.75 | 2.21 | 6.86 | 2.18 | 5.76 | 2.25 | 2.22 | 2.18 | 6 | 14 | 0.0011 |
|  | 虚伪(hypocrisy) | 2.73 | 1.40 | 5.10 | 2.04 | 5.55 | 1.88 | 5.09 | 2.32 | 4.55 | 2.82 | 11 | 6 | 0.0012 |
|  | 蛆虫(maggot) | 2.02 | 1.02 | 5.83 | 2.02 | 6.84 | 1.72 | 4.93 | 1.62 | 1.03 | 0.82 | 11 | 6 | 0.0006 |
|  | 粗鲁(rough) | 3.15 | 1.18 | 5.08 | 1.79 | 5.35 | 1.84 | 4.82 | 1.89 | 2.60 | 1.47 | 11 | 12 | 0.0006 |
|  | 下贱(nasty) | 2.61 | 1.28 | 5.60 | 2.15 | 5.86 | 2.05 | 4.03 | 1.95 | 4.63 | 2.52 | 3 | 9 | 0.0006 |
|  | 呕吐(vomit) | 2.38 | 1.33 | 5.23 | 2.38 | 6.23 | 1.83 | 5.23 | 2.02 | 2.48 | 0.61 | 7 | 6 | 0.0011 |
|  | 老鼠(rat) | 2.83 | 1.41 | 4.81 | 2.46 | 6.73 | 2.00 | 5.71 | 2.16 | 1.37 | 0.96 | 6 | 13 | 0.0026 |
|  | 腐烂(decay) | 2.46 | 1.34 | 5.02 | 2.46 | 5.94 | 1.97 | 5.43 | 2.33 | 3.67 | 1.67 | 14 | 9 | 0.0011 |

Table4. Rating results for neutral words.

| Neutral words | Valence | *SD* | Arousal | *SD* | Dominance | *SD* | familiarity | *SD* | concreteness | *SD* | First word  stroke number | Second word stroke number | lexical frequency |
| --- | --- | --- | --- | --- | --- | --- | --- | --- | --- | --- | --- | --- | --- |
| 钢铁(steel) | 5.38 | 1.37 | 3.30 | 1.54 | 5.40 | 2.20 | 5.01 | 2.19 | 1.55 | 1.00 | 9 | 10 | 0.0039 |
| 新闻(news) | 5.96 | 1.21 | 5.54 | 1.84 | 4.17 | 1.91 | 5.99 | 2.10 | 1.61 | 1.09 | 13 | 9 | 0.0153 |
| 激光(laser) | 5.32 | 1.33 | 4.44 | 2.07 | 4.45 | 1.87 | 4.55 | 1.97 | 3.21 | 2.59 | 16 | 6 | 0.0018 |
| 历史(history) | 5.60 | 1.50 | 3.89 | 1.76 | 3.21 | 2.08 | 5.15 | 2.29 | 4.00 | 2.83 | 4 | 5 | 0.0483 |
| 语言(language) | 5.53 | 1.28 | 3.54 | 1.55 | 6.45 | 1.72 | 6.31 | 2.07 | 3.42 | 3.36 | 9 | 7 | 0.0366 |
| 冰箱(refrigerator) | 5.50 | 1.34 | 3.40 | 1.68 | 6.53 | 1.85 | 5.98 | 2.10 | 1.25 | 0.91 | 6 | 15 | 0.0005 |
| 铁路(railway) | 5.10 | 1.15 | 3.43 | 1.93 | 4.38 | 2.01 | 5.36 | 2.24 | 1.63 | 1.26 | 10 | 13 | 0.0063 |
| 列车(train) | 5.33 | 1.12 | 3.45 | 1.75 | 4.44 | 2.20 | 6.10 | 2.03 | 1.63 | 1.34 | 6 | 4 | 0.0017 |
